# The role of *CRYAB* in tumor prognosis and immune infiltration: A Pan-cancer analysis

**DOI:** 10.3389/fsurg.2022.1117307

**Published:** 2023-01-13

**Authors:** Lang Cheng, Xiong Zou, Jiawei Wang, Jiange Zhang, Zengnan Mo, Houbao Huang

**Affiliations:** ^1^ Center for Genomic and Personalized Medicine, Guangxi key Laboratory for Genomic and Personalized Medicine, Guangxi Collaborative Innovation Center for Genomic and Personalized Medicine, Guangxi Medical University, Nanning, China; ^2^ Institute of Urology and Nephrology, The First Affiliated Hospital of Guangxi Medical University, Nanning, China; ^3^ Department of Urology, The First Affiliated Hospital of Wannan Medical College (Yijishan Hospital of Wannan Medical College), Wuhu, China; ^4^ Department of Urology, The Second Affiliated Hospital of Guangxi Medical University, Nanning, China

**Keywords:** *CRYAB*, prognosis, immune infiltration, tumor microenvironment, pan-cancer

## Abstract

**Purpose:**

There is evidence that the Crystallin Alpha B *(CRYAB)* gene is involved in the regulation of the tumor microenvironment and influences tumor prognosis in some cancers. However, the role of *CRYAB* gene in prognosis and immunology in pan-cancer is still unclear.

**Methods:**

In this study, we analyzed the transcriptional profiles and survival data of cancer patients from The Cancer Genome Atlas (TCGA) database. *CRYAB* gene and its relationships with pan-cancer were analyzed using R packages, TIMER2.0, GEPIA2, Sangerbox, UALCAN, cBioPortal, ESTIMATE algorithm, and STRING. Besides, real-time fluorescence quantitative polymerase chain reaction (RT-qPCR) was utilized to detect *CRYAB* expression in KIRC and a human KIRC cell line (Caki-1).

**Results:**

We found that *CRYAB* expression was different in tumors and adjacent tumors in human cancers, affecting patients’ prognosis in 15 cancer types. Additionally, *CRYAB* expression significantly correlated with tumor microenvironment (TME), immune checkpoints (ICP), tumor mutational burden (TMB), and microsatellite instability (MSI) in human cancers. Besides, *CRYAB* expression was positively associated with the immune infiltration of cancer-associated fibroblasts (CAFs) and endothelial cells in most human cancers. Based on enrichment analysis, the most prevalent *CRYAB* gene mechanism in malignant tumors may be through anti-apoptotic activity. Moreover, some FDA-approved drugs were found to be associated with *CRYAB* and might be potential cancer therapeutic candidates.

**Conclusions:**

*CRYAB* is a crucial component of the TME and influences immune cell infiltration, making it a promising biomarker to assess immune infiltration and prognosis in many malignancies.

## Introduction

With the rapid growth and aging of the global population, cancer and related deaths are increasing (1). Therefore, there is an urgent need for cancer management across the globe. Nonetheless, it is well known that tumors are difficult to conquer due to their heterogeneity, and because of the difference of origin location, cell type, and gene mutation form, tumors can show different morphology. These factors also affect the therapeutic effect of tumors. Although it has been confirmed that many genetic changes directly lead to phenotypic changes, the complex molecular mechanisms in many tumor lineages are still not fully elucidated (2). TCGA and Gene Expression Omnibus (GEO) analyzed a large number of human tumors to find molecular alterations at the DNA, RNA, protein, and epigenetic levels. The pan-cancer analysis uses these databases to find common features from different tumors. The analysis of molecular changes and their functions in different tumors will in the future tell us how to apply effective therapies to tumors with similar genetic phenotypes (3).

*CRYAB* comprises three domains: N-terminal, central, and C-terminal (4). As a molecular chaperone, the *CRYAB* gene could prevent damages induced by heat shock, radiation, and oxidative stress, thereby escaping cell apoptosis and promoting cell survival (5). Recent studies have confirmed that the *CRYAB* gene regulates tumor invasion and metastasis in colorectal cancer and gastric cancer (6, 7). Studies have shown that tumors with high expression of the *CRYAB* gene have a poor survival rate (8–10), implying that the *CRYAB* gene is a potential biomarker. However, the role of the *CRYAB* gene in pan-cancer prognosis has not been reported.

**Figure 1:**
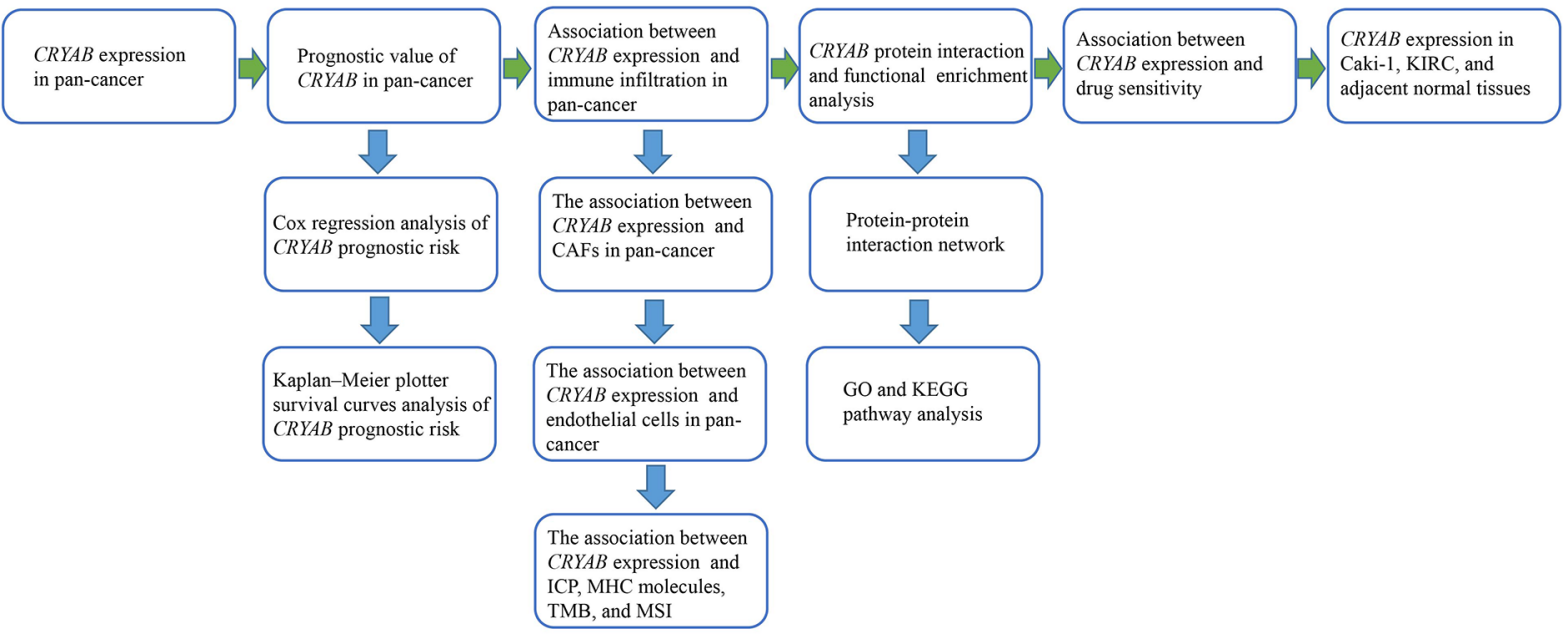
The flow chart of *CRYAB* in pan-cancer analysis.

*CRYAB* gene could suppress apoptosis of tumor cells and regulate the vascular endothelial growth factor (VEGF) causing tumorigenesis in breast cancer (11, 12). Besides, increasing evidence indicates that the *CRYAB* gene potentially modulates tumor development *via* the TME (13). One study (14) revealed that M2 macrophages in the TME promote lung cancer progression by upregulating *CRYAB* expression. Researchers (6) also discovered that the *CRYAB* gene increases colorectal cancer progression by inducing epithelial-mesenchymal transition (EMT). These studies implied that the *CRYAB* gene may have functional roles in M2 macrophages and EMT in lung cancer and colorectal cancer. With the development of tumor immunology and single-cell sequencing technology, researchers have proposed that tumor-associated macrophages play an important role in the development of cancer, and CAFs can induce EMT in human tongue squamous cell carcinoma (15–17). Tumor-associated macrophages, CAFs, and other tumor-infiltrating immune cells are important components of the tumor microenvironment. They are present in all stages of cancer and play a key role in the tumor development, progression, and metastasis (15, 18). *CRYAB*'s effect on immune infiltration will greatly affect cancer progression and needs to be taken seriously as part of pan-cancer research. Although there has been evidence of *CRYAB*'s effect on tumor prognosis and tumor-infiltrating lymphocytes, it has only been limited to a few tumors, and there has been no comprehensive pan-cancer analysis based on TCGA and GO. As such, we evaluated the function of the *CRYAB* gene in human tumors using a comprehensive pan-cancer analysis. We first evaluated the expression of *CRYAB* gene in pan-cancer to gain a general understanding of the gene function, and then investigated the effect of *CRYAB* expression on tumor prognosis and immune infiltration. In addition, we analyzed the molecular pathways of *CRYAB*-related genes and the drug sensitivity of *CRYAB* gene. Further, RT-qPCR was utilized to detect *CRYAB* expression in KIRC and Caki-1 to confirm the bioinformatics results. The process of this pan-cancer analysis is shown in Figure 1.

## Materials and methods

### Data acquisition and processing

We got the data from the TCGA *via* the UCSC Xena database (http://xena.ucsc.edu/), including transcriptional profiles and survival data. The R (version 4.1.3; The R Foundation for Statistical Computing, Vienna, Austria) language was used to process the original data. Online web tools were also used for data analysis.

### Expression analysis of CRYAB gene

To compare the expression of *CRYAB* in pan-cancer and their normal tissues, the TIMER2.0 database (http://timer.cistrome.org/) was employed. In the “Gene_DE” module, we entered the gene name “*CRYAB*” to obtain the results. Further, *CRYAB* gene expression was analyzed using the GTEx database as the control by the Sangerbox website (http://sangerbox.com). The relationship between *CRYAB* expression and the cancer pathological stages was examined using the “Stage Plot” module of GEPIA2 (http://gepia2.Cancer-pku.cn/). On the UALCAN database (http://ualcan.path.uab.edu/analysis.html) homepage, the “Clinical Proteomic Tumor Analysis Consortium (CPTAC) analysis” module was selected, then we entered the gene name “*CRYAB*” to explore the total protein and phosphoprotein of *CRYAB* protein in different cancers.

### Prognostic and survival analysis of CRYAB gene

We obtained survival data from the TCGA database. The data included complete follow-up records of patients and *CRYAB* expression profiles. Then, the Kaplan-Meier method and log-rank test were used for survival analysis (*p* < 0.05). The median expression of the *CRYAB* gene was selected as the cut-off value for the human cancer dichotomy, hence categorizing patients into high and low risk groups. Subsequently, the R package “survminer” and “survival” were used to plot the survival curve. Cox analysis was also performed using the R package “survival” and “forestplot”. Lastly, we obtained respective outcomes of overall survival (OS), disease-specific survival (DSS), disease-free interval (DFI), and progression-free interval (PFI).

### Correlation analysis of CRYAB expression with immune infiltration

*CRYAB* expression and immune infiltration in human malignancies were investigated using the “Immune-Gene” module of TIMER2.0 (http://timer.cistrome.org/). The algorithms in the above analysis process included QUANTISEQ, TIDE, XCELL, MCPCOUNTER, EPIC, TIMER, and CIBERSORT. The *P*-values and partial correlation (cor) values were calculated by Spearman's rank correlation test.

### Correlation analysis of CRYAB expression with immune checkpoints (ICP), tumor mutational burden (TMB), and microsatellite instability (MSI)

SangerBox website (http://sangerbox.com) was used to explore the association between *CRYAB* expression and immune checkpoints (ICP), tumor mutational burden (TMB), and microsatellite instability (MSI) in human tumors. In the “Gene+” module of the Sangerbox website, we entered the gene name “*CRYAB*”, and got the results by using the Pearson correlation test.

### Enrichment analysis of CRYAB-related gene

On the website of STRING (https://cn.string-db.org/), we obtained the top 50 *CRYAB*-binding proteins. First, the gene name “*CRYAB*” was entered and the organism of “Homo sapiens” was searched. Then the parameters of “the full STRING network”, “evidence”, “experiments”, “low confidence (0.150)”, and “no more than 50 interactors” were set. On the GEPIA2 website, the top 200 *CRYAB*-correlated targeting genes were obtained from the “Similar Gene Detection” module by setting TCGA tumor and TCGA normal. These two datasets were integrated for gene ontology (GO) enrichment and Kyoto encyclopedia of genes and genomes (KEGG) pathway analysis *via* R-package “org.Hs.eg.db”, “clusterProfiler”, and “enrichplot”. Lastly, The R package “ggplot2″ was used to realize the visualization of enrichment paths.

### Drug sensitive analysis

RNA expression data and drug data from the NCI-60 cell line were obtained from the CellMiner database (https://discover.nci.nih.gov/cellminer/home.do). To ensure the reliability of the analysis results, 792 drugs that have passed clinical trials are and FDA approved were selected. We used Pearson correlation to explore the association between *CRYAB* expression and drug sensitivity, and *p* < 0.05 was considered statistically significant.

### Clinical sample collection

We collected seven specimens of renal clear cell carcinoma and their adjacent noncancerous tissues from The Second Affiliated Hospital of Guangxi Medical University. The Ethics Committee of The Second Affiliated Hospital of Guangxi Medical University approved the work (Approval Number: 2021-KY(0123)). The study participants signed the informed consent forms.

### Cell culture

Both the human KIRC cell line (Caki-1) and the human renal cortex proximal tubule epithelial cells (HK-2) were bought from the American Type Culture Collection. Caki-1 was cultured in McCoy's 5A medium (Procell, Wuhan, China) with 10% fetal bovine serum (Gibco, Thermo Fisher Scientific, United States) and placed in an incubator set at 37°C and 5% CO_2_. HK-2 was cultured in Minimum Essential Medium (MEM, Procell, Wuhan, China) with 10% fetal bovine serum (Gibco, Thermo Fisher Scientific, United States) and placed in an incubator set at 37°C and 5% CO_2_.

### RT-qPCR analysis

TRIzol (Beyotime, Shanghai, China) was used to extract total RNA from the tissues and cells. The OD260/280 values were used to detect the total RNA concentration and purity. FastKing gDNA Dispelling RT SuperMix cDNA synthesis Kit (TIANGEN, Beijing, China) was used to synthesize cDNAs, and at the same time, it was used to remove the residual genomes. Polymerase Chain Reaction was performed by using FastStart Essential DNA Green Mster (Roche Diagnostics GmbH, Mannheim, Germany) and LightCycler® 96 Instrument (Roche Diagnostics GmbH, Mannheim, Germany). All experiments were repeated three times. GAPDH was used as an internal standard, and the primer sequences of GAPDH were 5′–GGAGTCCACTGGCGTCTTCA –3′ (Forward Primer) and 5′ –GTCATGAGGCCAGAAATGAAGG– 3′ (Reverse Primer). The primer sequences of *CRYAB* were 5′ –AGGTGTTGGGAGATGTGATTGA– 3′ (Forward Primer) and 5′ –GGATGAAGTAATGGTGAGAGGGT– 3′ (Reverse Primer). We used the 2^−*ΔΔ*Ct^ method to calculate the relative expression of *CRYAB*.

### Statistical analysis

Statistical analyses were performed using GraphPad Prism 8 (GraphPad Software, United States). The paired T-test was used to analyze the relative expression of *CRYAB* in tumor tissues and adjacent non-tumor tissues. The Student's *t*-test was used to analyze the relative expression of *CRYAB* in Caki-1 and HK-2*. P *< 0.05 was considered statistically significant.

## Results

### CRYAB gene expression analysis

**Figure 2:**
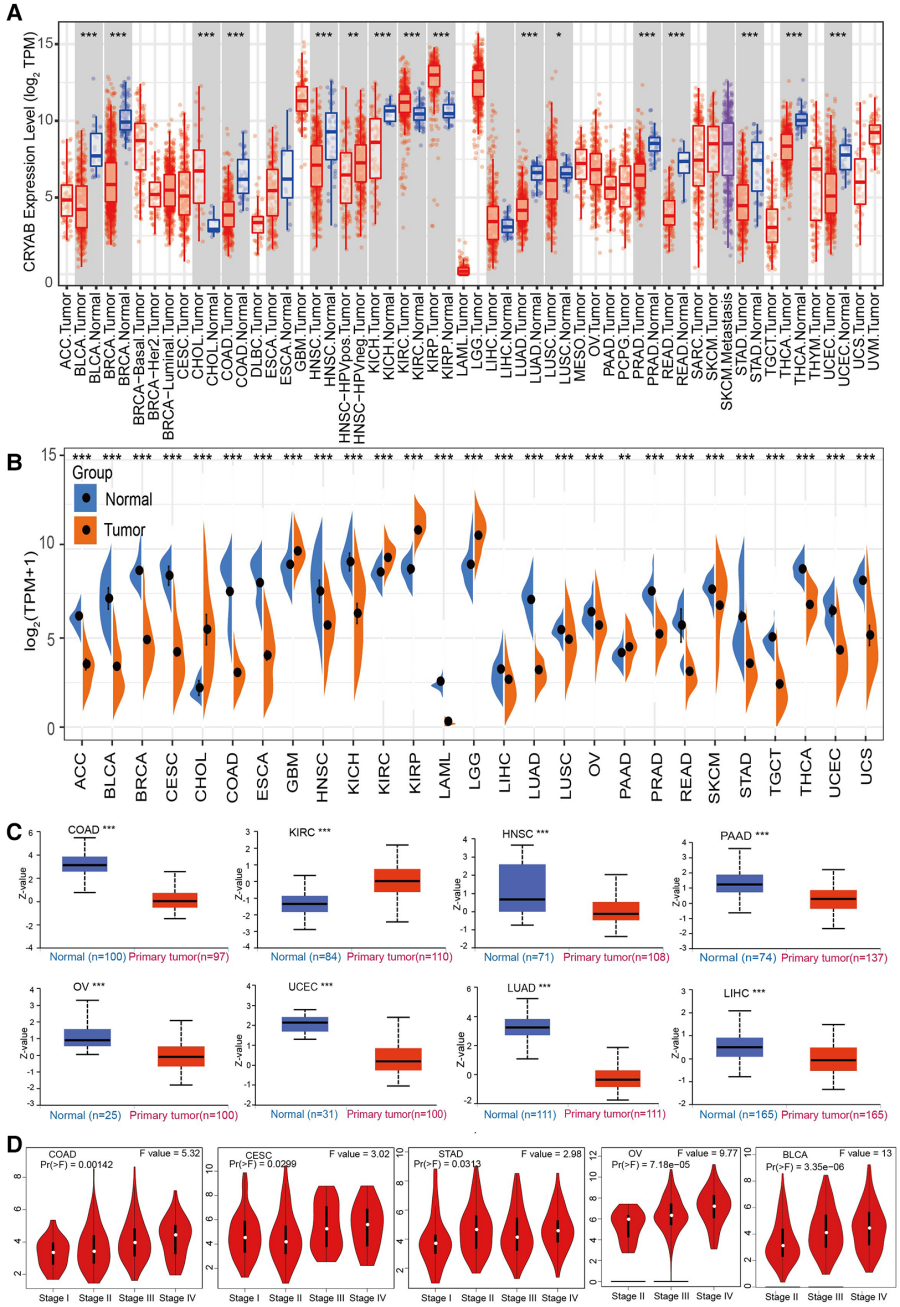
The expression profile of *CRYAB* in pan-cancer. (**A**) TIMER2.0 analysis of the *CRYAB* gene expression in pan-cancer. (**B**) Sangerbox analysis of *CRYAB* gene expression in pan-cancer in TCGA. GTEx database served as the control. (**C**) *CRYAB* protein expression in COAD, KIRC, HNSC, PAAD, OV, UCEC, LUAD, and LIHC in the CPTAC database. (**D**) The correlation between *CRYAB* and cancer pathological stages in COAD, CESC, STAD, OV, and BLCA as determined using GEPIA2 tool. *p < 0.05 **p < 0.01; ***p < 0.001.

The results of TIMER2.0 revealed lower *CRYAB* gene expression in bladder urothelial carcinoma (BLCA), breast invasive carcinoma (BRCA), colon adenocarcinoma (COAD), head and neck squamous cell carcinoma (HNSC), kidney chromophobe (KICH), lung adenocarcinoma (LUAD), lung squamous cell carcinoma (LUSC), prostate adenocarcinoma (PRAD), rectum adenocarcinoma (READ), stomach adenocarcinoma (STAD), thyroid carcinoma (THCA), and uterine corpus endometrial carcinoma (UCEC) than that in adjacent normal tissues except for cholangiocarcinoma (CHOL), kidney renal clear cell carcinoma (KIRC), and kidney renal papillary cell carcinoma (KIRP) (Figure 2A). Several tumors in TCGA did not have paired normal tissue. The GTEx database was used as a control to further investigate *CRYAB* gene expression. As shown in Figure 2B, *CRYAB* gene expression was lower in tumors than that in normal tissues except for CHOL, glioblastoma multiforme (GBM), KIRC, KIRP, brain lower grade glioma (LGG), and pancreatic adenocarcinoma (PAAD). Besides, we analyzed the *CRYAB* total protein expression in cancers *via* CPTAC and found that, unlike normal tissues, *CRYAB* protein expression levels were significantly low in COAD, HNSC, PAAD, OV, UCEC, LUAD, and liver hepatocellular carcinoma (LIHC) except for KIRC (Figure 2C). Further, we examined the relationship between *CRYAB* gene expression and pathological stages of cancers. *CRYAB* gene was significantly upregulated in the advanced pathological stage of COAD, cervical squamous cell carcinoma and endocervical adenocarcinoma (CESC), STAD, ovarian serous cystadenocarcinoma (OV), and BLCA (*P* < 0.05) (Figure 2D).

### Survival analysis of CRYAB in human tumors

**Figure 3:**
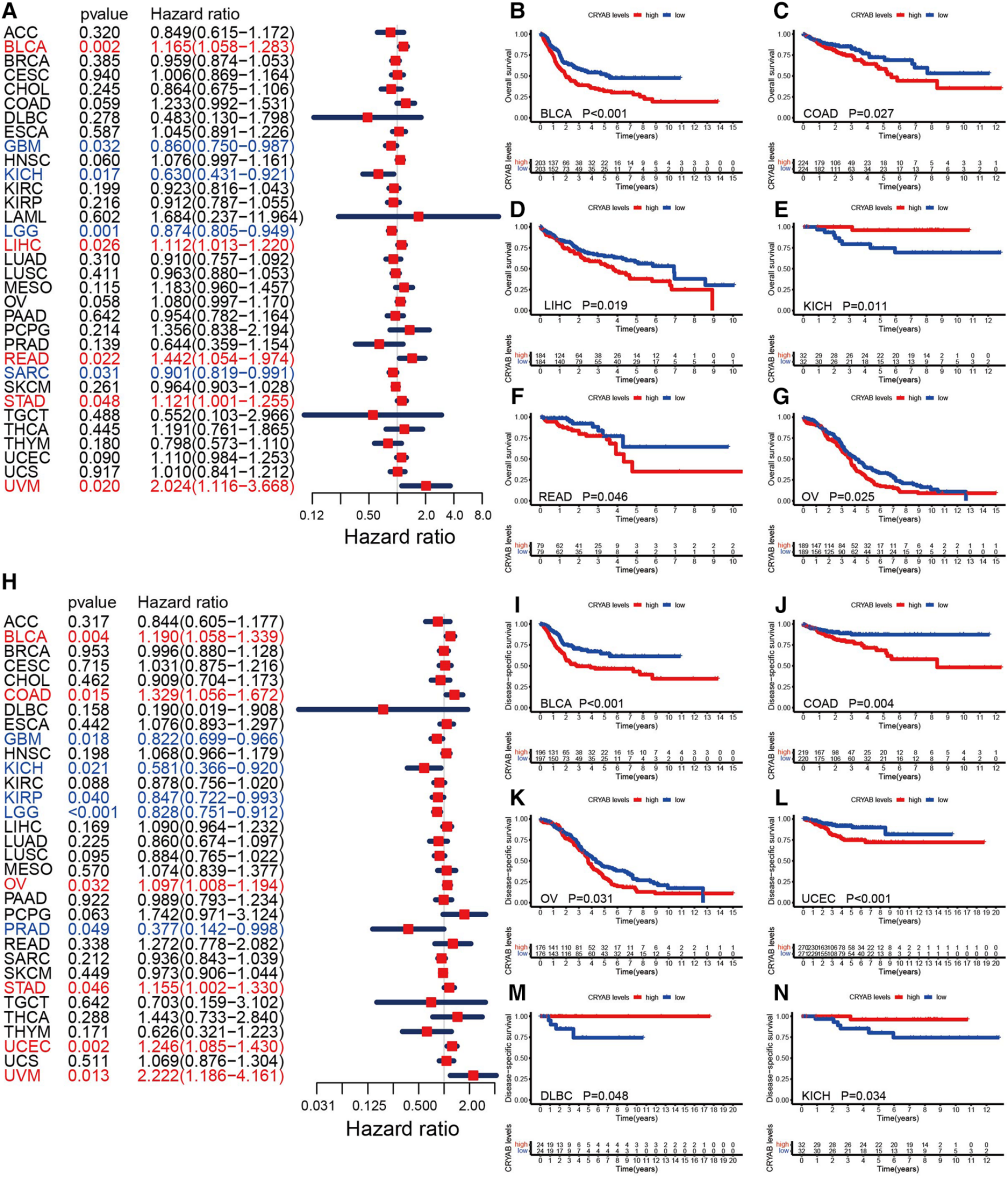
Relationship between *CRYAB* and OS and DSS in pan-cancer in TCGA database. (**A**) Cox model analysis of *CRYAB* for OS in pan-cancer. (**B–G**) The Kaplan–Meier plotter survival analysis of *CRYAB* for OS. (**H**) Cox model analysis of *CRYAB* for DSS in pan-cancer. (**I–N**) The Kaplan–Meier plotter survival analysis of *CRYAB* for DSS.

To understand the prognostic significance of the *CRYAB* gene, we analyzed its relevance to survival outcomes in human tumors, including OS, DFI, PFI, and DSS. First, the OS of the *CRYAB* gene was analyzed using Cox proportional hazards model. Subsequently, we plotted Kaplan–Meier plotter survival curves for cancers with significant prognosis in the TCGA database. Figure 3A showed the results of the Cox proportional hazards model analysis, indicating that the *CRYAB* gene is a risk factor in BLCA, LIHC, READ, STAD, and UVM, yet a protective factor in GBM, KICH, LGG, and SARC. Cancer patients were categorized into low and high *CRYAB* expression groups. The Kaplan–Meier method analysis showed that high-*CRYAB* groups were linked to the poor OS in BLCA (Figure 3B), COAD (Figure 3C), LIHC (Figure 3D), READ (Figure 3F), and OV (Figure 3G), but with a positive prognosis in KICH (Figure 3E). Afterward, the DSS was analyzed based on the data for tumor death in the TCGA database. In the forest plot (Figure 3H), we noted that the *CRYAB* gene was a risk factor in BLCA, COAD, OV, STAD, UCEC, and UVM, but a protective factor in GBM, KICH, KIRP, LGG, and PRAD. Kaplan–Meier method indicated that high *CRYAB* expression was remarkably related to poor DSS in BLCA (Figure 3I), COAD (Figure 3J), OV (Figure 3K), and UCEC (Figure 3L), but with an optimistic prognosis in lymphoid neoplasm diffuse large B-cell lymphoma (DLBC) (Figure 3M) and KICH (Figure 3N).

**Figure 4:**
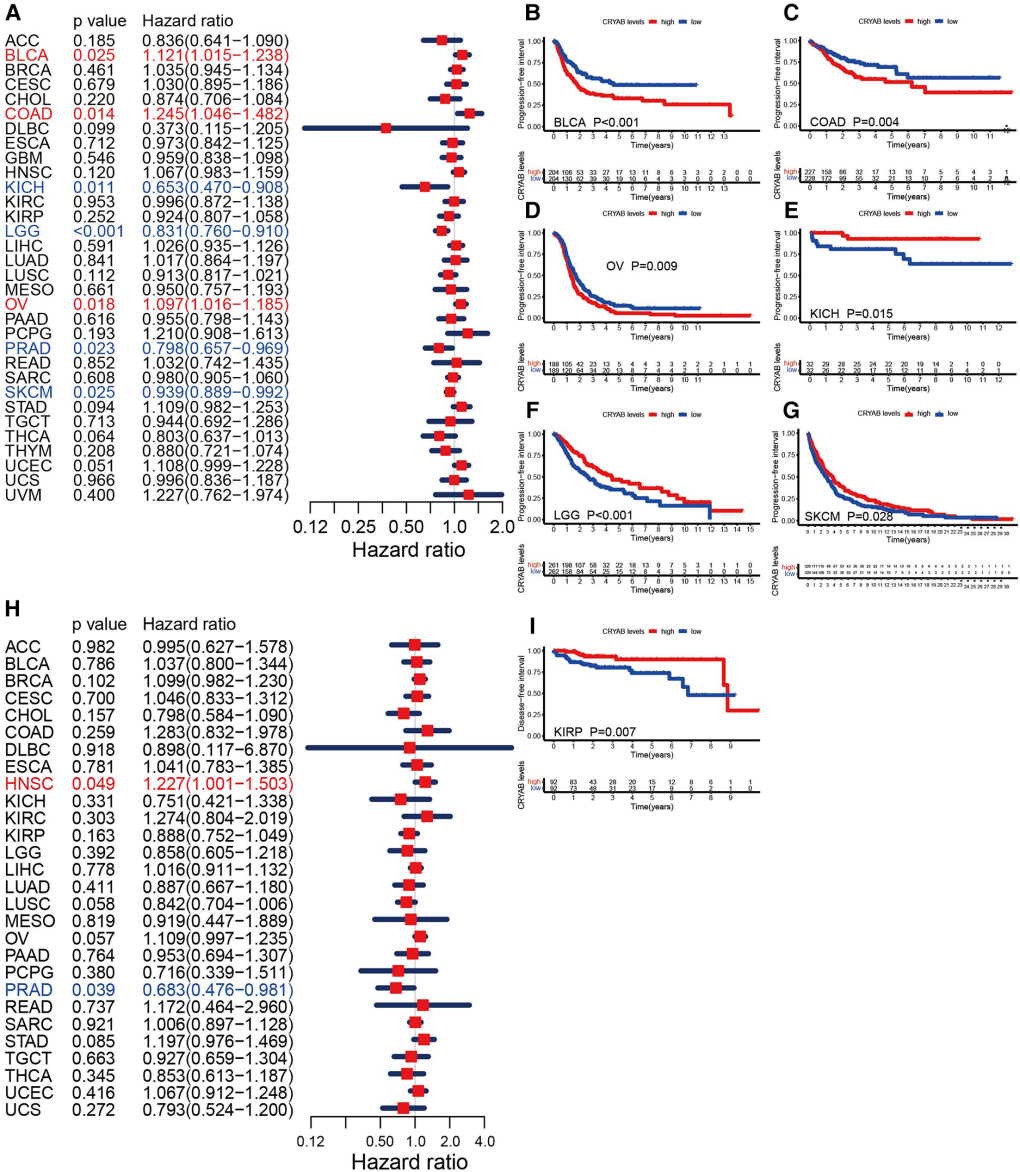
Relationship between *CRYAB* and PFI and DFI in pan-cancer in TCGA database. (**A**) Cox model analysis of *CRYAB* for PFI in pan-cancer. (**B–G**) The Kaplan–Meier plotter survival analysis of *CRYAB* for PFI. (**H**) Cox model analysis of *CRYAB* for DFI in pan-cancer. (**I**) The Kaplan–Meier plotter survival analysis of *CRYAB* for DFI in KIRP.

Furthermore, we investigated the association between the *CRYAB* gene expression and PFI. The forest plots showed that the *CRYAB* gene was a risk factor in BLCA, COAD, and OV, but a protective factor in KICH, LGG, PRAD, and skin cutaneous melanoma (SKCM) (Figure 4A). According to the Kaplan-Meier method, high *CRYAB* expression was linked to poor prognosis in BLCA (Figure 4B), COAD (Figure 4C), and OV (Figure 4D); however, high *CRYAB* expression was related to optimistic prognosis in KICH (Figure 4E), LGG (Figure 4F), and SKCM (Figure 4G). We also analyzed the effect of *CRYAB* gene on DFI of cancer patients. Based on forest plot results, the *CRYAB* gene was a risk factor in HNSC, but a protective factor in PRAD (Figure 4H). According to the Kaplan-Meier analysis of DFI, high *CRYAB* expression was related to an optimistic prognosis in KIRP (Figure 4I). The above results confirmed that *CRYAB* gene expression was closely linked to the clinical outcomes of various cancers.

### Association between CRYAB expression and immune infiltration in human tumors

**Figure 5:**
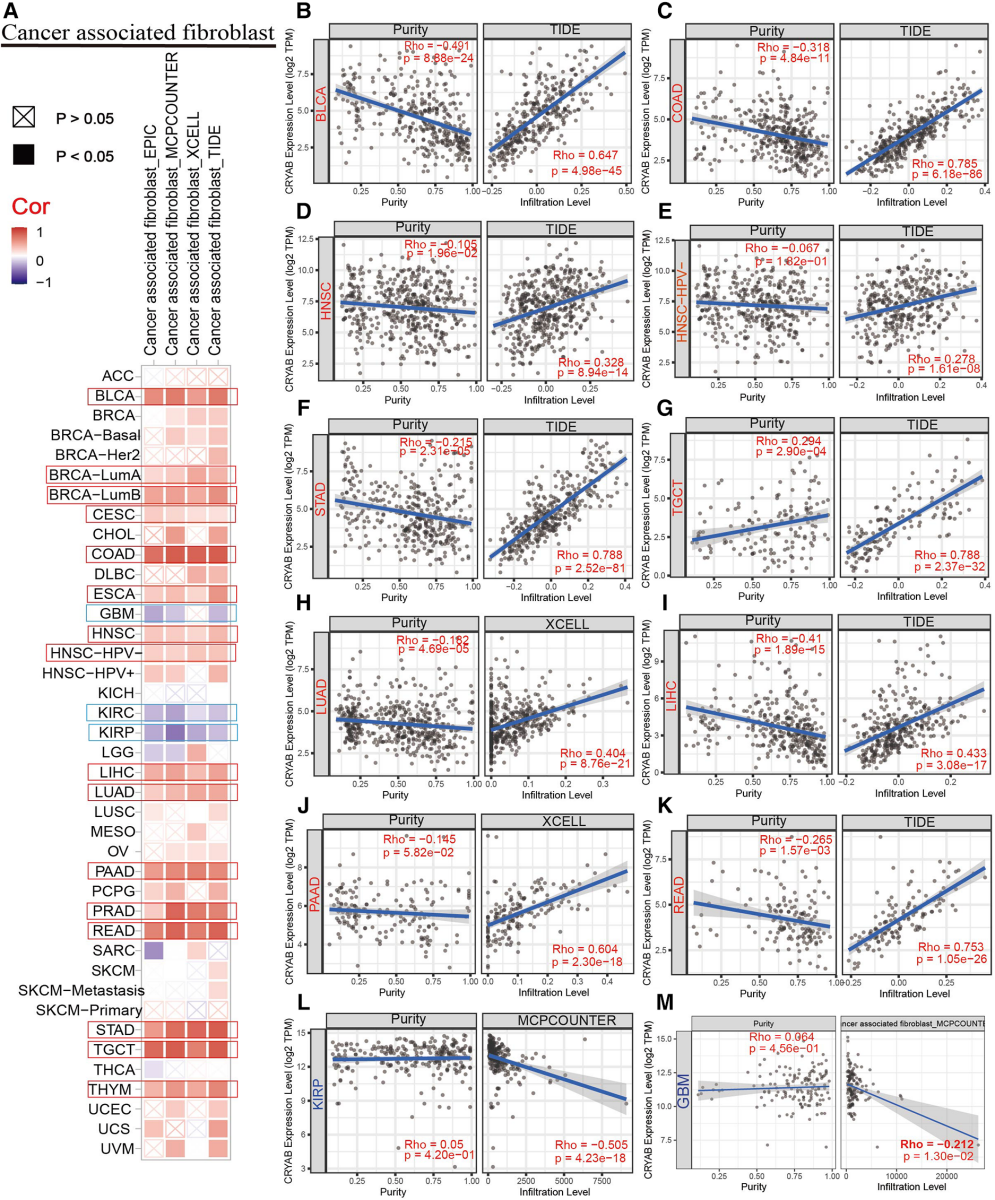
Association between *CRYAB* expression and CAFs infiltration in pan-cancer. (**A**) A summary of using different algorithms to explore the associations between *CRYAB* expression and CAFs infiltration in pan-cancer. The *CRYAB* gene positively related to CAFs in BLCA (**B**), COAD (**C**), HNSC (**D**), HNSC-HPV- (**E**), STAD (**F**), TGCT (**G**), LUAD (**H**), LIHC (**I**), PAAD (**J**), and READ (**K**), but negatively related to CAFs in KIRP(**L**) and GBM(**M**).

**Figure 6:**
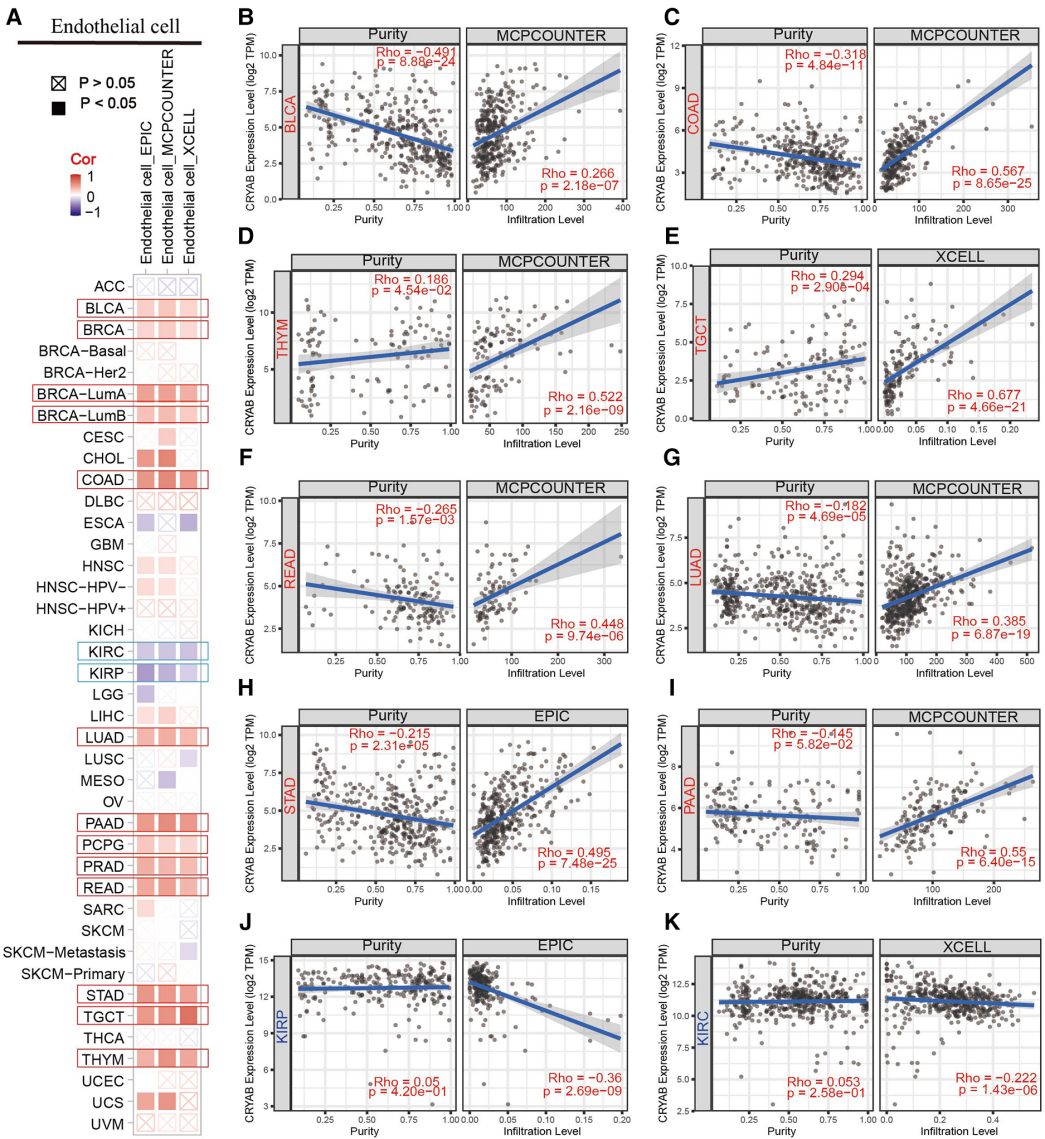
Association between *CRYAB* expression and infiltration of endothelial cells in pan-cancer. (**A**) A summary of using different algorithms to explore the associations between *CRYAB* expression and endothelial cells infiltration in pan-cancer. The *CRYAB* gene positively correlated with endothelial cells in BLCA (**B**), COAD (**C**), THYM (**D**), TGCT (**E**), READ (**F**), LUAD (**G**), STAD (**H**), and PAAD (**I**) but negatively correlated with endothelial cells in KIRP (**J**) and KIRC (**K**).

TIMER2.0 was used to investigate the extent of immune infiltration and *CRYAB* gene expression in human cancers (Figure 5A, Figure 6A, and Supplementary Figure S1). Consequently, the *CRYAB* gene positively related to CAFs in BLCA, COAD, HNSC, HNSC-HPV-, STAD, TGCT, LUAD, LIHC, PAAD, READ, BRCA-LumA, BRCA-LumB, ESCA, CESC, PRAD, and THYM (Figure 5B–K, Supplementary Figures S1A–F), but negatively related to CAFs in KIRP, GBM, and KIRC (Figure 5L–M, Supplementary Figure S1l). Also, the *CRYAB* gene positively correlated with endothelial cells in BLCA, COAD, THYM, TGCT, READ, LUAD, STAD, PAAD, BRCA, BRCA-LumA, BRCA-LumB, PCPG, and PRAD (Figure 6B–I, Supplementary Figures S1G–K), but negatively associated with their counterparts in KIRP and KIRC (Figure 6J–K).

### Association between CRYAB expression and immune checkpoints (ICP), major histocompatibility complex (MHC) molecules, tumor mutational burden (TMB), and microsatellite instability (MSI) in human tumors

**Figure 7:**
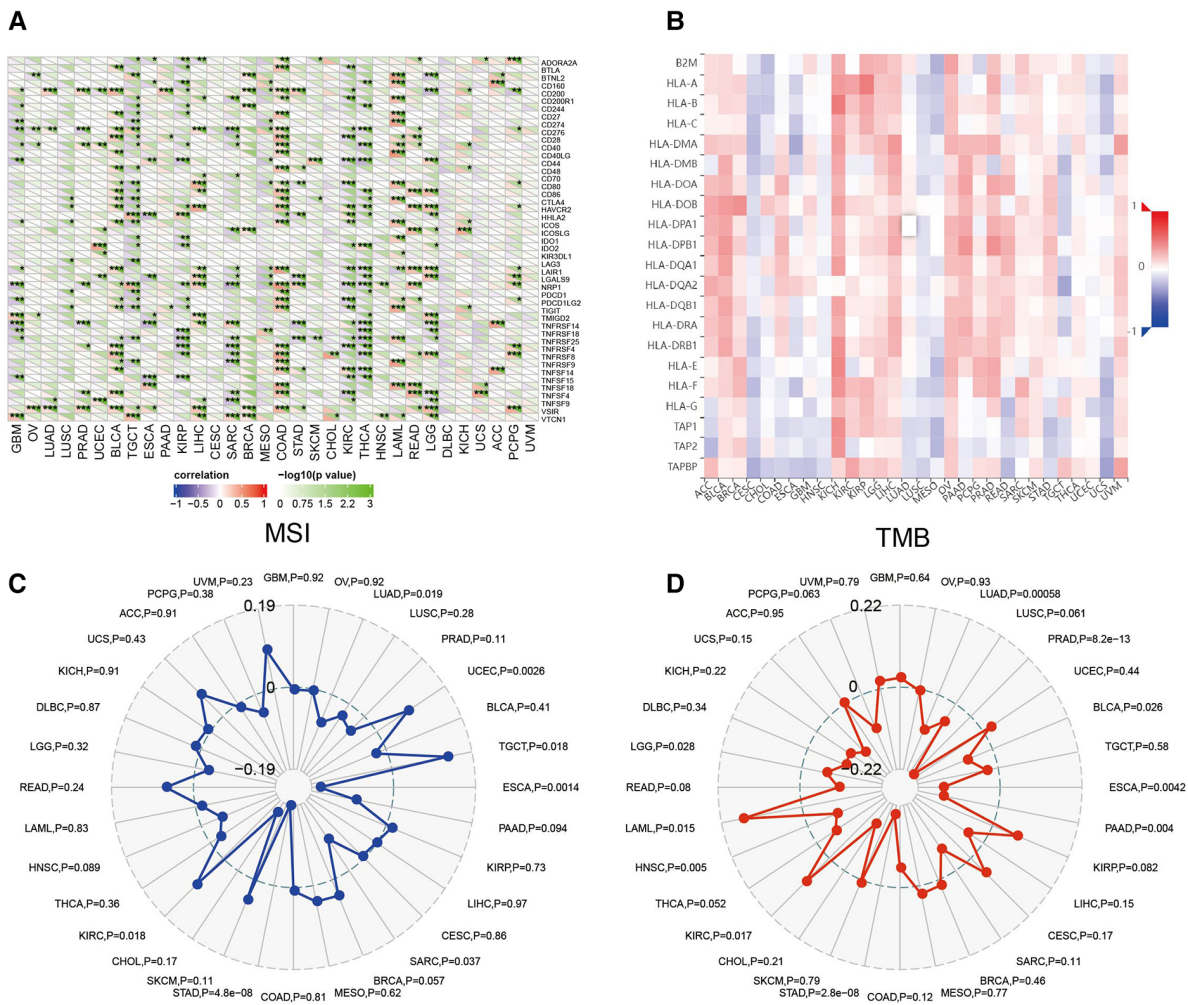
Association between *CRYAB* expression and immune checkpoints (ICP) (**A**), major histocompatibility complex (MHC) molecules (**B**), microsatellite instability (MSI) (**C**), and tumor mutational burden (TMB) (**D**) in human tumors.

Sangerbox website was used to evaluate the association between *CRYAB* expression and immune checkpoint (ICP) genes. As shown in Figure 7A, 47 immune checkpoint genes were evaluated, and *CRYAB* expression was associated with immune checkpoint genes in different cancers, including BLCA, TGCT, COAD, KIRP, KIRC, SARC, THCA, (acute myeloid leukemia) LAML, READ, and LGG. In BLCA, COAD, LAML, READ, and LGG, *CRYAB* expression was positively associated with immune checkpoint genes. In TGCT, KIRP, SARC, KIRC, and THCA, *CRYAB* expression was negatively correlated with immune checkpoints. Using the TISIDB website, the association between *CRYAB* expression and 21 MHCs were analyzed. *CRYAB* expression was positively associated with 21 MHCS in BLCA, BRCA, KICH, LIHC, OV, and UVM (Figure 7B).

In TME, MSI and TMB are predictors of tumor immunotherapy efficacy (19, 20). Sangerbox website was used to evaluate the association between *CRYAB* expression and MSI/TMB. *CRYAB* expression was positively associated with MSI in UCEC, TGCT, and KIRC, while negatively associated with MSI in LUAD, ESCA, SARC, and STAD (Figure 7C). Moreover, *CRYAB* was positively associated with TMB in KIRC and LAML, but negatively correlated with LUAD, PRAD, BLCA, ESCA, PAAD, STAD, and LGG (Figure 7D).

### Enrichment analysis of CRYAB-related genes

**Figure 8:**
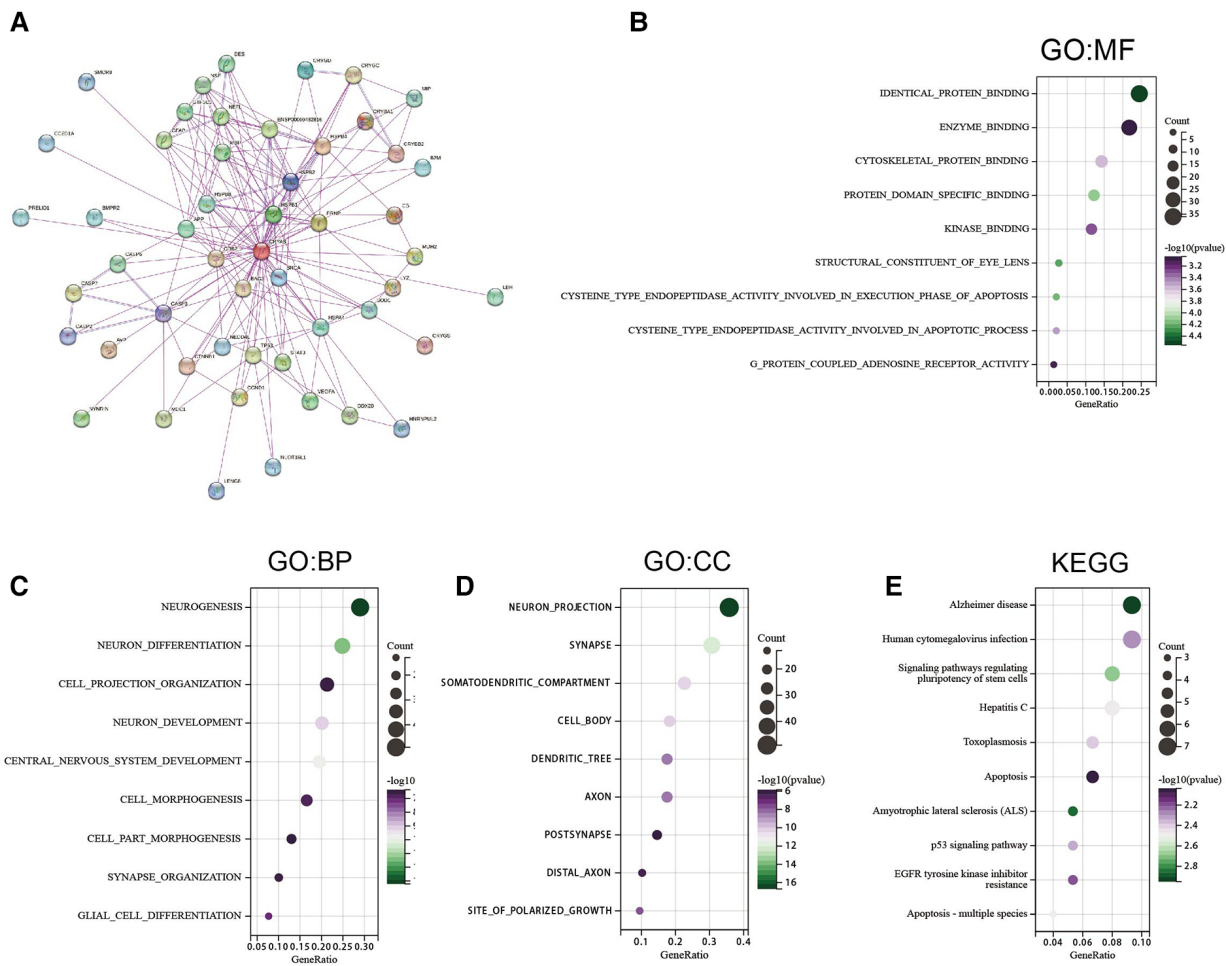
Gene enrichment analysis. (**A**) 50 *CRYAB*-binding proteins experimentally verified to interact with *CRYAB*. (**B**) GO molecular functions of *CRYAB*-binding and interacting genes (**C**) GO biological processes associated with *CRYAB*-binding and interacting genes. (**D**) GO cellular components associated with *CRYAB*-binding and interacting genes. (**E**) KEGG pathway analysis of *CRYAB*-binding and related genes.

Pathway enrichment analysis of *CRYAB*-related genes and *CRYAB* binding proteins was conducted to understand the mechanisms by which the *CRYAB* gene regulates cancer cells on a molecular level. We screened 50 *CRYAB* binding proteins and their protein-protein interaction (PPI) network using the STRING database (Figure 8A). The top 200 *CRYAB*-related genes were picked using GEPIA2 from the TCGA database. Based on these two datasets, we used the R package “clusterProfiler”, “org.Hs.eg.db”, and “enrichplot” for functional enrichment analysis. Based on the GO analysis, The major molecular functions of *CRYAB* included “identical protein binding”, “cysteine type endopeptidase activity involved in the execution phase of apoptosis”, and “cysteine_type endopeptidase activity involved in the apoptotic process” (Figure 8B). The major biological functions of *CRYAB* included “neurogenesis”, “neuron differentiation”, and “apoptotic process” (Figure 8C). Cell components related to *CRYAB* gene included “neuron projection”, “synapse”, and “cell_cell junctions” (Figure 8D). The KEGG results revealed that *CRYAB*-related genes enriched in “Alzheimer”s disease”, “Human cytomegalovirus infection”, “Hepatitis C”, “Apoptosis”, “Signaling pathways regulating pluripotency of stem cells”, “EGFR tyrosine kinase inhibitor resistance”, “Apoptosis—multiple species”, and “p53 signaling pathway” (Figure 8E). These results revealed that the *CRYAB* gene might be involved in regulating tumor immune microenvironment and tumor growth.

### Association between CRYAB expression and drug sensitivity

**Figure 9:**
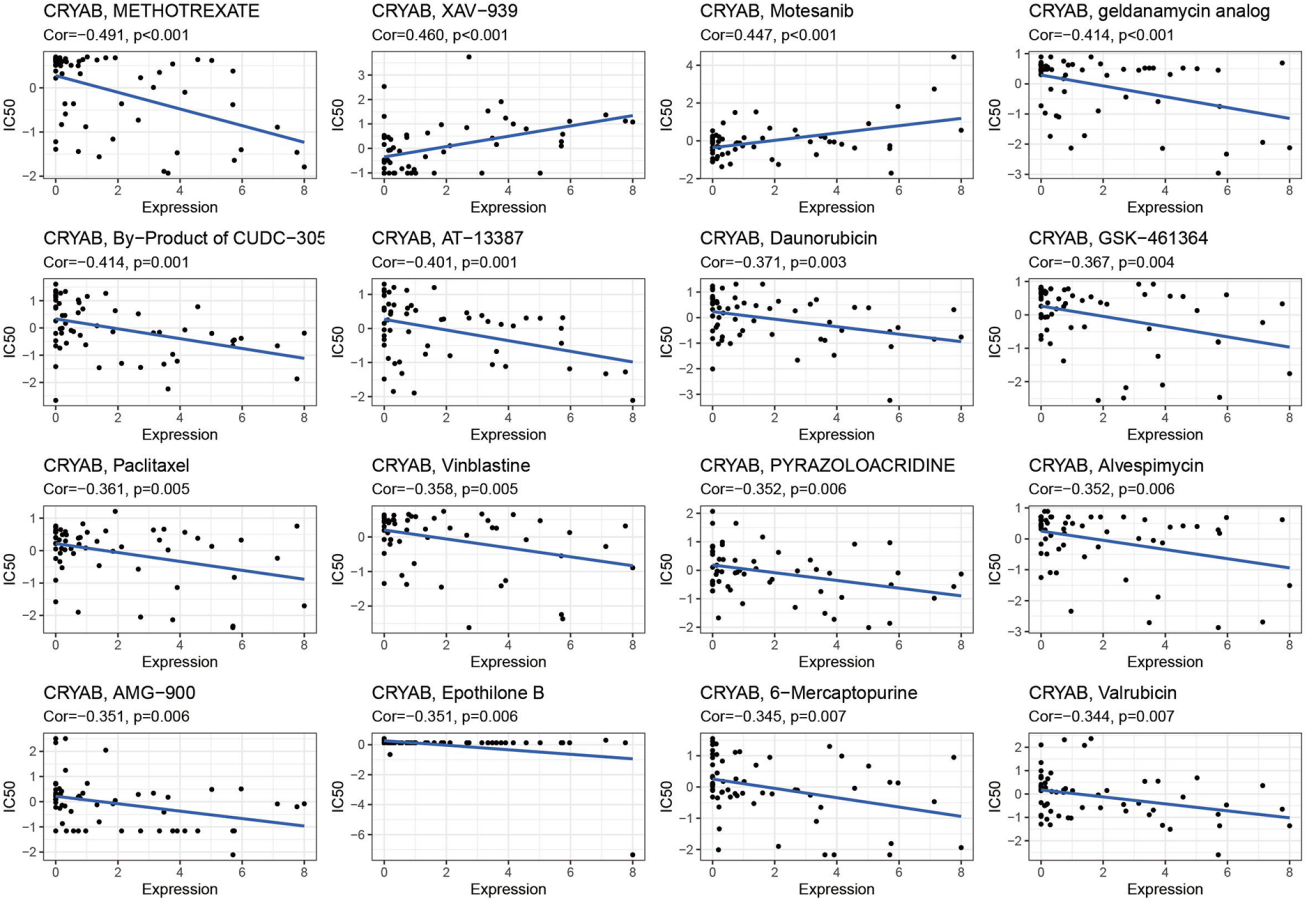
Scatter plot of the association between *CRYAB* expression and drug sensitivity.

The detection of *CRYAB* expression in NCI-60 cell lines showed that *CRYAB* expression was associated with drug sensitivity (Supplementary Table S1). With the increase of *CRYAB* expression, the IC50 of METHOTREXATE, geldanamycin analog, By−Product of CUDC−305, AT-13387, Daunorubicin, GSK−461364, Paclitaxel Vinblastine, PYRAZOLOACRIDINE, Alvespimycin, AMG−900, Epothilone B, 6−Mercaptopurine, and Valrubicin decreased in cancer cells (Figure 9). This indicated that the *CRYAB* expression developed tumor cell sensitivity to these drugs. By contrast, it decreased the drug sensitivity of XAV−939 and Motesanib (Figure 9).

### Validation of CRYAB expression in kidney renal clear cell carcinomas and caki-1

**Figure 10:**
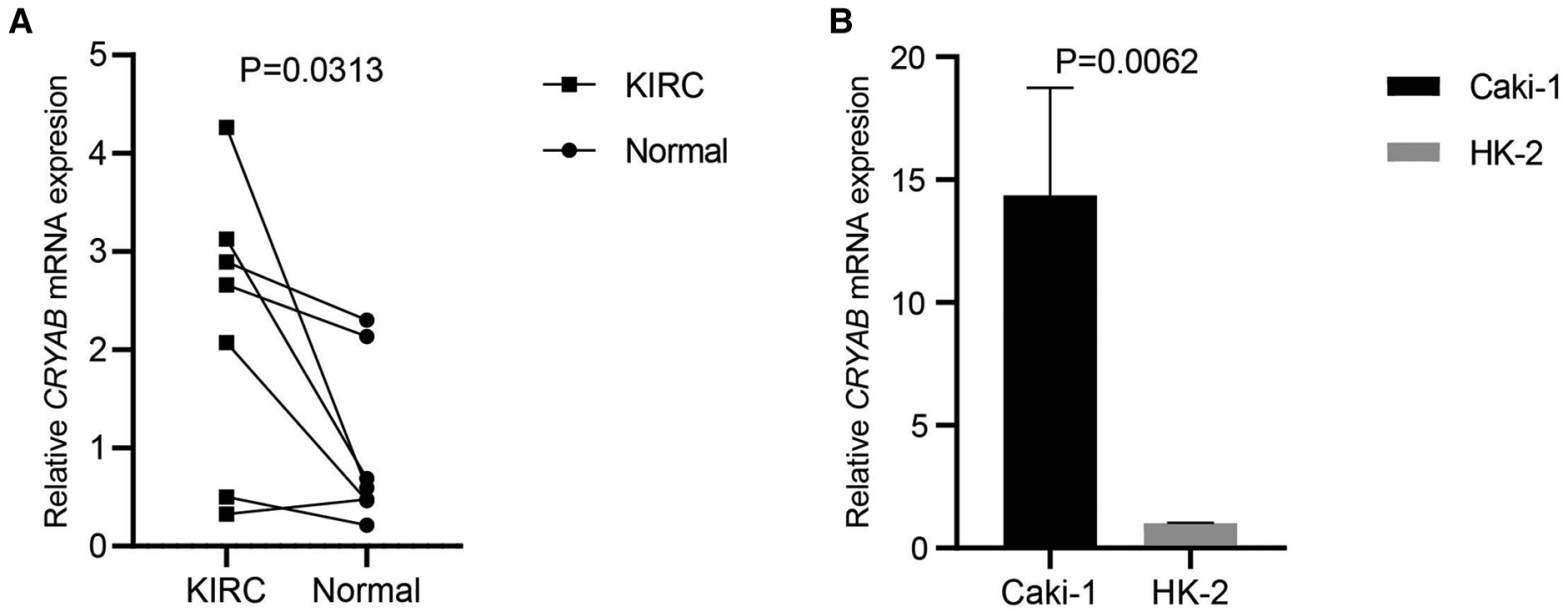
*CRYAB* expression in KIRC and caki-1. (**A**) Seven specimens of KIRC and their adjacent normal tissues were used to detect *CRYAB* expression. *CRYAB* mRNA expression was significantly elevated in KIRC tissues compared with normal tissues. (**B**) *CRYAB* mRNA expression was elevated in Caki-1 compared with HK-2.

To detect *CRYAB* expression in KIRC and Caki-1, we collected the specimens of renal clear cell carcinoma and their adjacent noncancerous tissues and cultured Caki-1 and HK-2 for RT-qPCR. *CRYAB* mRNA expression was significantly higher in KIRC tissues than that in normal tissues (*P* < 0.05, Figure 10A). Besides, *CRYAB* mRNA expression was elevated in Caki-1 compared with HK-2 (*P* < 0.05, Figure 10B).

## Discussion

The incidence of patients with cancer is globally increasing with rapid growth and the aging population (1). A number of these cancers have an aggressive clinical presentation with a poor prognosis. Notably, it is critical to foresee the clinical outcome of tumor patients when developing a treatment plan. TNM staging is adopted to evaluate the clinical outcome of patients. Nonetheless, TNM staging is not significantly precise. For instance, although early diagnosis and advanced therapeutic interventions have shown progress in COAD, the five-year survival rate remains at approximately 45% (21). Molecular biomarkers improve the probability of precisely predicting cancer prognosis (22). Therefore, analyzing molecular biomarkers can significantly benefit most cancer patients by helping in choosing the appropriate treatment approach. Previous studies have shown that cancer patients with high levels of *CRYAB* demonstrated an unfavorable survival (8–10), indicating its potential as a prognostic biomarker. As a molecular chaperone, the *CRYAB* gene could prevent protein degradation caused by cell stress stimulation (including heat shock, oxidative stress, or radiation) (23). A previous study revealed that *CRYAB* blocks apoptosis by directly interacting with caspase-3, Bax, and Bcl-xS, thereby suppressing their translocation to mitochondria (24, 25). Besides, the anti-inflammatory pathway of *CRYAB* involves immunomodulatory responses *via* endosomal/phagosomal CD14 and Toll-like receptors 1 and 2 (26). However, the immunological effect of the *CRYAB* gene in pan-cancer background warrants additional research. Herein, we identified the role of the *CRYAB* gene in human cancers *via* a pan-cancer analysis of TCGA and CPTAC databases.

First, this work analyzed *CRYAB* expression in pan-cancer using the TIMER2.0. The expression of *CRYAB* gene in most cancers was lower than their adjacent normal tissues except for CHOL, KIRC, and KIRP. Further, we explored *CRYAB* gene expression in pan-cancer *via* the Sangerbox tool using the TCGA and GTEx databases. Consequently, the expression of *CRYAB* was lower in tumor tissues than in healthy tissues except for CHOL, GBM, KIRC, KIRP, LGG, and PAAD. These findings were consistent with previous studies in BLCA, OV, KIRC, COAD, HNSC, and GBM, PRAD (27–33). In this pan-cancer analysis, we found that *CRYAB* gene expression was elevated in KIRC through TCGA and GTEx database. Based on our previous studies on KIRC, we want to further verify whether the expression of *CRYAB* in our clinical samples is consistent with that in TCGA. So we selected seven clinical samples of KIRC from our hospital and a human KIRC cell line to verify *CRYAB* expression through RT-qPCR. *CRYAB* mRNA expression was significantly elevated in KIRC tissues and Caki-1. The above results were in line with the bioinformatics analysis results in KIRC.

Furthermore, the *CRYAB* protein expression levels were analyzed in cancers through CPTAC. As a result, *CRYAB* expression levels were lower in COAD, UCEC, HNSC, LIHC, LUAD, PAAD, and OV than in healthy tissues except for KIRC. The results in COAD, HNSC, LUAD, and OV were consistent with that of previous studies (29–31, 34). Based on the above findings, it appears that the protein levels matched the RNA levels.

Subsequently, we examined the relationship between *CRYAB* gene expression and pathological stages of cancers. The results showed that *CRYAB* expression was upregulated with tumor stage in COAD, CESC, STAD, OV, and BLCA; other reports are in agreement with the above results in BLCA and COAD (21, 27).

The effects of the *CRYAB* gene were evaluated on the survival outcome of pan-cancer. The Cox proportional hazards model analysis of OS indicated that the *CRYAB* gene is a risk factor in BLCA, LIHC, READ, STAD, and UVM, while a protective factor in GBM, KICH, LGG, and SARC. The Kaplan–Meier method analysis revealed that high-*CRYAB* groups correlated with poor OS in BLCA, COAD, LIHC, OV, and READ, but with optimistic an prognosis in KICH. The Cox proportional hazards model analysis of DSS showed that the *CRYAB* gene was a risk factor in BLCA, COAD, OV, STAD, UCEC, and UVM, but a protective factor in GBM, KICH, KIRP, LGG, and PRAD. The Kaplan-Meier analysis revealed that high-*CRYAB* groups were linked to poor DSS in BLCA, COAD, OV, and UCEC, but with a positive prognosis in DLBC and KICH. Nonetheless, the endpoint of OS potentially reduces the feasibility of clinical research. Non-cancer-related deaths do not necessarily indicate tumor biology, aggressiveness, or response to therapy. Additionally, OS or DSS warrants longer follow-up. Therefore, the use of PFI or DFI can more effectively indicate the effect of various factors on patients. We examined the PFI or DFI of the *CRYAB* gene in cancer patients using the Cox proportional hazards model and Kaplan-Meier analysis. The Cox proportional hazards model analysis of PFI revealed that the *CRYAB* gene was a risk factor in BLCA, COAD, and OV, but a protective factor in KICH, LGG, PRAD, and SKCM. According to the Kaplan-Meier method, high-*CRYAB* groups were associated with poor PFI in BLCA, COAD, and OV, but with optimistic prognosis in KICH, LGG, and SKCM. On the other hand, high-*CRYAB* groups were linked to optimistic DFI in KIRP. Based on forest plot results, the *CRYAB* gene was a risk factor in HNSC, but a protective factor in PRAD. Current studies show that the *CRYAB* gene is an oncogene, and patients with high expression of *CRYAB* are in the advanced tumor stage or have a poor prognosis. Juliane Volkmann et al. (30) discovered that the *CRYAB* gene is unexpressed or only weakly expressed in 75% of ovarian cancer samples, whereas high *CRYAB* expression correlates with poor outcomes (OS, PFS) in ovarian cancer patients. *CRYAB* expression in COAD was similar to that in OV and high expression of *CRYAB* positively correlated with high-grade malignancies, more advanced tumors, lymph nodes, metastatic (TNM) cancer stages, and poor survival outcomes (21, 29). In one bioinformatics analysis of BLCA, the results revealed that patients demonstrated poor OS and DFS when they expressed high levels of *CRYAB* (27). In head and neck cancer, Chin David et al. (31) revealed that *CRYAB* is an independent marker for poor survival outcomes, further emphasizing its potential role as a prognostic biomarker. So far, the *CRYAB* gene has been shown to suppress tumorigenesis only in prostate cancer and nasopharyngeal carcinoma (NPC) (33, 35). In the nude mice model, *CRYAB* activation potentially inhibits the occurrence of nasopharyngeal carcinoma. High *CRYAB* gene expression improves NPC progression (35). Besides, downregulated *CRYAB* expression was noted in patients with PRAD, serving as a protective gene for PRAD (33), which is consistent with our results. In this study, high *CRYAB* expression was linked to poor OS and DSS in BLCA, COAD, LIHC, READ, STAD, UCEC, and UVM patients, but with optimistic OS and DSS in GBM, KICH, KIRP, LGG, SARC, and PRAD patients. Meanwhile, high *CRYAB* expression was linked to poor DFI and PFI in BLCA, COAD, OV, and HNSC patients, but with optimistic DFI and PFI in KICH, LGG, PRAD, PRAD, and SKCM patients. Given that *CRYAB* expression has several promising interactions in eukaryotic cells, it might be beneficial to one cancer yet harmful to another (36), and with a distinct molecular mechanism of *CRYAB* in cancer development (25).

TME innately modulates cancer progression, allowing tumor cells to evade immune surveillance, thereby significantly influencing immunotherapy outcomes and survival prognosis of patients (13). TME comprises infiltrating immune cells, and cancer-related stromal cells, among other normal epithelial cells. Accumulating evidence suggests that stromal cells regulate the occurrence and development of many tumors (37–40). Therefore, we further investigated the relationship between *CRYAB* expression and the immune infiltration of cancer-related stromal cells and immune cells. In our study, the *CRYAB* gene positively correlated with CAFs in most cancers but negatively related to CAFs in KIRP, GBM, and KIRC. Moreover, we identified a positive correlation between the *CRYAB* gene and endothelial cells in the majority of cancers, but a negative correlation in KIRC and KIRP. We noted the first potential relationship between CAFs and *CRYAB* expression in tumors.

Of note, CAFs are a primary component of TME. In addition to their interaction with cancer cells, CAFs affect extracellular matrix (ECM), immune infiltration, and other elements of the TME (15). Histopathological analysis has shown that CAFs play a role in the prognosis of different cancers (41, 42). At present, CAFs exert a tumor-promoting effect in a majority of cancers. Patients with high levels of CAFs infiltration have a poor prognosis (43). Also, CAFs promote angiogenesis by producing abundant pro-angiogenic factors including VEGFA, PDGFC, FGF2, osteopontin, and secreting frizzled associated protein 2 (SFRP2). CAFs potentially inhibit or impair CD8+T cells' function by secreting TGF*β*, increasing arginase activity, expressing abundant FAS ligand, or killing CD8+ T cells in an antigen-specific manner (15, 44–46). In many cancers, the infiltration of CD8+ T cells is associated with better clinical outcomes, but endothelial cells negatively correlate with survival (47). As such, whether CAFs promoted endothelial cells and suppressed CD8 + T cells, or regulated each other, remains to be explored.

Analysis of data on pathological stages of cancer revealed a possible connection between high *CRYAB* expression and advanced pathological staging in CESC, COAD, BLCA, and STAD due to the increased infiltration of CAFs and endothelial cells in the TME. Examination of prognostic data showed a potential association between high *CRYAB* expression and poor prognosis in BLCA, COAD, HNSC, LIHC, READ, and STAD. This was ascribed to the increased infiltration of CAFs and endothelial cells, and decreased CD8+ T cells infiltration in the TME. In contrast, high expression of the *CRYAB* gene in GBM and KIRP reduced infiltration of CAFs and endothelial cells, which improved the survival outcome. In conclusion, our findings demonstrated that *CRYAB* may influence the clinical outcomes of cancer patients by influencing CAFs and endothelial cells infiltration in tumors. CAFs may play a major role in mediating the effect of *CRYAB* on the clinical outcome of cancer patients. This association needs to be further studied.

In our study, the association between *CRYAB* expression and ICP, TMB, and MSI in human tumors also confirmed that *CRYAB* closely associated with TME in human cancers. Besides, we found that *CRYAB* was negatively related to TMB and MSI in some cancers, such as LUAD, PRAD, BLCA, ESCA, PAAD, STAD, SARC, and LGG. This meant *CRYAB* may influence immunotherapy response in these cancers. Moreover, we explored the association between *CRYAB* expression and drug sensitivity in cancer cell lines. The results indicated that *CRYAB* expression developed tumor cell sensitivity to a number of chemotherapeutic drugs, such as METHOTREXATE, geldanamycin analog, By−Product of CUDC−305, AT-13387, Daunorubicin, GSK−461364, Paclitaxel Vinblastine, PYRAZOLOACRIDINE,.

Alvespimycin, AMG−900, Epothilone B, 6−Mercaptopurine, and Valrubicin. By contrast, it decreased the drug sensitivity of XAV−939 and Motesanib. Among these drugs, Motesanib is often used in bladder cancer to develop the antitumor effect of cisplatin (48). METHOTREXATE can be used to treat many kinds of cancer, such as LUAD, HNSC, and BRCA (49–51). The above results indicated that *CRYAB* expression could influence drug response and it could serve as a potential drug target.

To explore the possible mechanism underlying the effects of *CRYAB* on cancer patients, we conducted GO and KEGG enrichment analyses of binding proteins and genes related to *CRYAB* expression. The results indicated that *CRYAB*-related genes enriched in “Alzheimer”s disease”, “Human cytomegalovirus infection”, “Hepatitis C”, “Apoptosis”, “Signaling pathways regulating pluripotency of stem cells”, “EGFR tyrosine kinase inhibitor resistance”, “Apoptosis—multiple species”, and “p53 signaling pathway”. The “EGFR tyrosine kinase inhibitor resistance” of KEGG results was consistent with the drug sensitivity results. In our study, we found *CRYAB* decreased the drug sensitivity of Motesanib which is a tyrosine kinase inhibitor. These findings expand our understanding of the role of *CRYAB* in human tumors. Studies have shown that *CRYAB* is an anti-apoptotic protein that negatively regulates the expression of pro-apoptotic proteins (24, 52). In OV, high expression of *CRYAB* suppressed tumor necrosis factor-related apoptosis-inducing ligand (TRAIL) and promoted tumor growth (30). *CRYAB* enhanced tumorigenesis by modulating VEGF and increased resistance to anti-VEGF therapy in breast cancer (11, 12). Altogether, these findings show that *CRYAB* has complex roles in malignant tumors. The most notable effect of *CRYAB* is its anti-apoptosis activity in tumors. Moreover, *CRYAB* may affect the prognosis of patients by influencing CAFs infiltration levels in tumors.

In this study, we performed a comprehensive analysis to evaluate the function of the *CRYAB* gene in human tumors, but there are still some limitations in this experiment. First, we used TCGA and GTEx datasets for pan-cancer analysis, but the data of some special tumors were still not available. Second, in this study, we mainly carried out bioinformatics analysis. The potential function of *CRYAB* needs to be verified *in vivo*/*in vitro* experiments. Third, even though we demonstrated that *CRYAB* may influence the prognosis of cancer patients, specifically targeting infiltration of CAFs and endothelial cells in TME. However, the exact pathway by which *CRYAB* is involved in immune regulation requires further investigation.

In conclusion, this is arguably the first pan-cancer analysis of the *CRYAB* gene. *CRYAB* gene expression varied in different cancers and their adjacent normal tissues. In addition, *CRYAB* is a crucial component of the TME and influences immune cell infiltration, making it a promising biomarker to assess immune infiltration and prognosis in many malignancies. Furthermore, *CRYAB* has potential impacts on cellular biological processes and molecular pathways in tumors. Besides, a number of FDA-approved drugs were found to be associated with *CRYAB* and might be potential cancer therapeutic candidates. This careful analysis provides valuable insights into the role of the *CRYAB* gene in prognosis and immunology in human tumors as well as new evidence for accurate and customized cancer therapy.

## Data Availability

The original contributions presented in the study are included in the article/**Supplementary Material**, further inquiries can be directed to the corresponding author/s.
