# Supplementary material for: The role of CRYAB in tumor prognosis and immune infiltration: A Pan-cancer analysis
Source: Front Surg. 2023 Jan 13;9:1117307. doi: 10.3389/fsurg.2022.1117307 (PMC9880180; doi:10.3389/fsurg.2022.1117307)
Supplement: Supplementary file 1 [file Datasheet1.docx]

**Supplementary Materials**

**Table 1:** Association between *CRYAB* expression and drug sensitivity.

| Gene | Drug | cor | pvalue |
| --- | --- | --- | --- |
| *CRYAB* | METHOTREXATE | -0.4912 | 6.75E-05 |
| *CRYAB* | XAV-939 | 0.459642 | 0.00022 |
| *CRYAB* | Motesanib | 0.447476 | 0.000337 |
| *CRYAB* | geldanamycin analog | -0.41435 | 0.000997 |
| *CRYAB* | By-Product of CUDC-305 | -0.41353 | 0.001023 |
| *CRYAB* | AT-13387 | -0.40116 | 0.00149 |
| *CRYAB* | Daunorubicin | -0.37122 | 0.0035 |
| *CRYAB* | GSK-461364 | -0.36702 | 0.00392 |
| *CRYAB* | Paclitaxel | -0.36139 | 0.004554 |
| *CRYAB* | Vinblastine | -0.35804 | 0.004973 |
| *CRYAB* | PYRAZOLOACRIDINE | -0.35224 | 0.005778 |
| *CRYAB* | Alvespimycin | -0.35194 | 0.005823 |
| *CRYAB* | AMG-900 | -0.35067 | 0.006016 |
| *CRYAB* | Epothilone B | -0.35055 | 0.006034 |
| *CRYAB* | 6-Mercaptopurine | -0.34462 | 0.00701 |
| *CRYAB* | Valrubicin | -0.34392 | 0.007133 |
| *CRYAB* | 6-(4-pyrimidinyl)-1H-indazole derivative | 0.336067 | 0.008659 |
| *CRYAB* | Tegafur | -0.33385 | 0.009137 |
| *CRYAB* | Docetaxel | -0.33348 | 0.009221 |

**
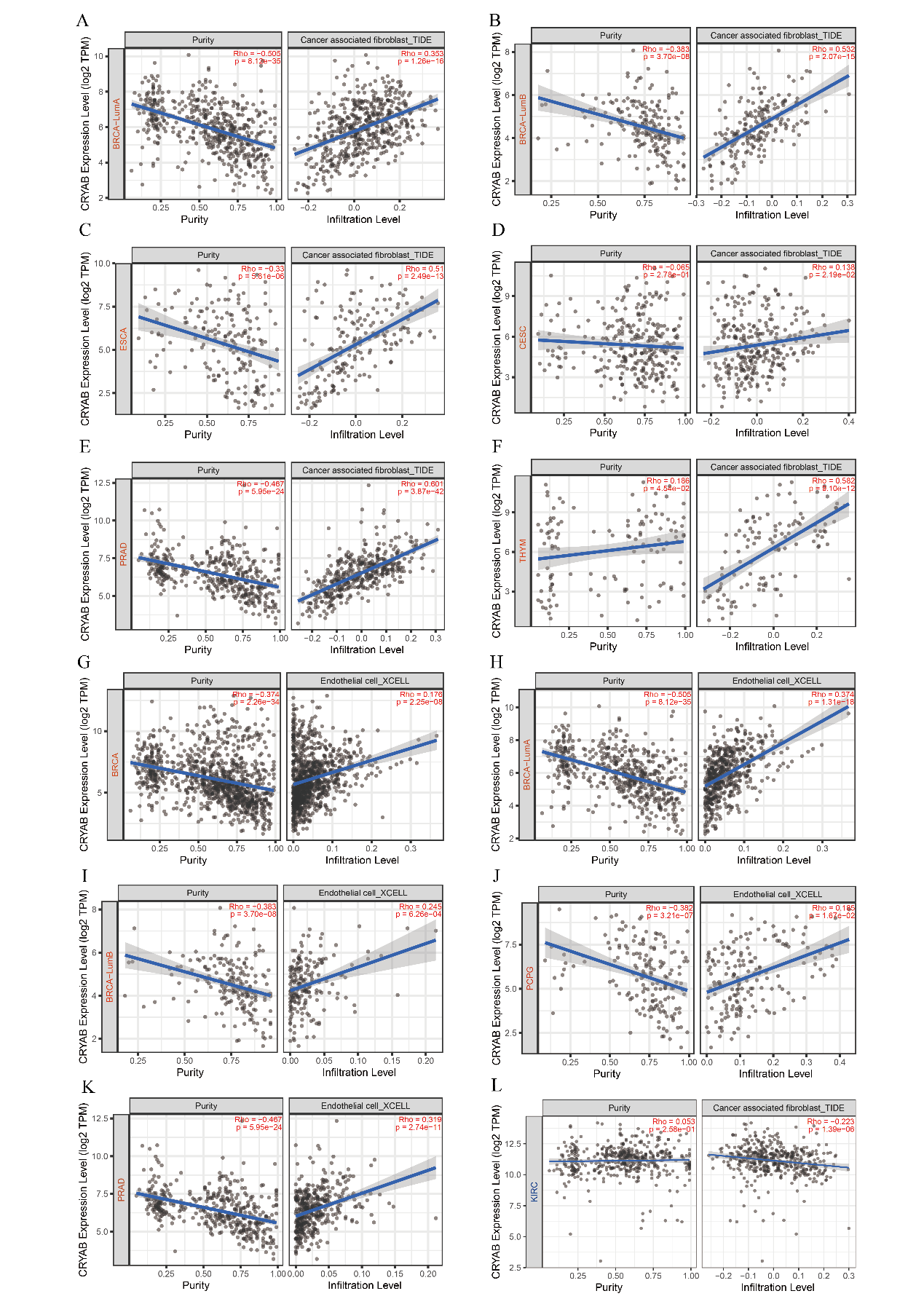
**

**Figure S1:** Association between *CRYAB* with the infiltration of CAFs and endothelial cells in different cancers. The *CRYAB* gene promoted CAFs in BRCA-LumA (A), BRCA-LumB (B), ESCA (C), CESC (D), PRAD (E), and THYM (F), but inhibited CAFs in KIRC (L). The *CRYAB* gene promoted endothelial cells in BRCA (G), BRCA-LumA (H), BRCA-LumB (I), PCPG (J), and PRAD (K).
